# Determinants of outcome in atrial functional tricuspid regurgitation

**DOI:** 10.1186/s44156-026-00140-4

**Published:** 2026-10-19

**Authors:** Jawza Aldakhil, Kush Patel, Sveeta Badiani, Steffen E. Petersen, Guy Lloyd, Sanjeev Bhattacharyya

**Affiliations:** 1 Heart Valve Clinic & Echocardiography Laboratory, Barts Heart Centre, St Bartholomew’s Hospital, St Bartholomew’s Hospital, West Smithfield, London, EC1A 7BE UK; 2 NIHR Barts Biomedical Research Centre, William Harvey Research Institute, Queen Mary University London, Charterhouse Square, London, EC1M 6BQ UK; 3 Institute of Cardiovascular Sciences, UCL, London, UK; 4 Cleveland Clinic London, London, UK; 5 Barts Heart Centre, St Bartholomew’s Hospital, Barts Health NHS Trust, West Smithfield, London, EC1A 7BE UK; 6 College of Applied Medical Sciences, King Saud Bin Abdulaziz University for Health Sciences, Riyadh, Saudi Arabia; 7 King Abdullah International Medical Research Centre, Riyadh, Saudi Arabia

**Keywords:** Atrial functional tricuspid regurgitation, Atrial fibrillation, Echocardiography, Right atrial strain

## Abstract

**Background:**

Atrial functional tricuspid regurgitation (AFTR) is increasingly recognized as a result of atrial remodelling in patients with atrial fibrillation (AF). However, prognostic predictors in this population remain incompletely defined.

**Objectives:**

To evaluate echocardiographic characteristics and identify predictors of all-cause mortality in patients with AFTR.

**Methods:**

We retrospectively studied consecutive patients with AF who underwent transthoracic echocardiography between 2017 and 2025. Inclusion required significant right atrial remodelling, defined as a right atrial area > 25 cm^2^. Patients with at least moderate functional tricuspid regurgitation comprised the AFTR cohort, while patients with mild/trivial/no tricuspid regurgitation served as controls. Patients with primary valvular disease, cardiomyopathy, congenital heart disease, device-related valve disease, RV dilatation, or more than mild RV systolic dysfunction were excluded. Cox proportional hazards regression was performed to identify predictors of mortality.

**Results:**

A total of 152 patients were included, comprising 120 patients with AFTR and 32 controls. Compared with controls, patients with AFTR demonstrated larger atrial dimensions, higher right-sided hemodynamic-burden, impaired RV longitudinal strain, and reduced reservoir RA strain. During a median follow-up of 6.3 years, 53 deaths (45%) occurred among patients with AFTR. In univariable Cox analysis, age (HR 1.02, 95% CI 1.00-1.04; *p* = 0.016), TR Vmax (HR 1.59, 95% CI 1.07–2.35; *p* = 0.020), and lower reservoir RA strain (HR 0.90, 95% CI 0.84–0.96; *p* = 0.003) were associated with mortality. In multivariable analysis, reservoir RA strain remained independently associated with mortality (adjusted HR 0.88, 95% CI 0.81–0.95; *p* < 0.001).

**Conclusion:**

AFTR is characterized by marked biatrial remodelling and impaired right atrial function. Reduced RA reservoir strain was independently associated with mortality, supporting a potential role for RA deformation imaging in risk stratification in AFTR.

## Background

Atrial Fibrillation (AF) is the most common cardiac arrhythmia. The prevalence is 2% in the general population, and it increases with age to 10–12% in those over the age of 80 years [1]. Chronic AF leads to atrial enlargement and tricuspid annular dilatation, resulting in secondary tricuspid regurgitation [2] with about a quarter of all patients with long-standing AF developing either mitral or tricuspid regurgitation.

Atrial Functional Tricuspid Regurgitation (AFTR) is characterised by tricuspid regurgitation secondary to right atrial and tricuspid annular dilatation in the setting of chronic AF and preserved right ventricular geometry and function [3, 4]. Severe AFTR is associated with symptoms, heart failure hospitalisations, and adverse clinical outcomes [5].

Atrial strain imaging can detect atrial dysfunction and remodelling before chamber dilatation occurs [6], and may provide important prognostic information in cardiovascular disease [7]. However, data evaluating echocardiographic predictors of outcomes in AFTR, particularly the role of right atrial strain, remain limited. Therefore, this study aimed to evaluate structural and functional echocardiographic characteristics and identify predictors of mortality in patients with AFTR.

## Methods

Patients with atrial fibrillation who underwent transthoracic echocardiography between 1 January 2017 and 31 June 2025 were screened. Patients were eligible if they demonstrated significant atrial remodelling, defined for this study as right atrial area > 25 cm^2^ and at least moderate functional tricuspid regurgitation in the absence of primary tricuspid valve disease, RV dilatation, or more than mild RV systolic dysfunction. Control subjects consisted of patients with atrial fibrillation but mild/trivial/no mitral or tricuspid regurgitation irrespective of right atrial size.

Valve regurgitation aetiology was determined using comprehensive clinical and echocardiographic assessment. Patients with primary (organic) valvular disease were excluded, including leaflet prolapse or flail, rheumatic disease, endocarditis, congenital valve abnormalities, prior valve surgery or transcatheter intervention, and cardiac device implantation. Patients with other significant valvular disease (including aortic regurgitation or stenosis, mitral stenosis, tricuspid stenosis, pulmonary regurgitation, or pulmonary stenosis), cardiomyopathy, congenital heart disease, or inadequate image quality were also excluded.

Patient demographics and clinical characteristics were extracted from electronic care records. This included age, sex, body surface area, atrial fibrillation subtype, cardiovascular risk factors including diabetes mellitus, hypertension, hyperlipidaemia, smoking status, coronary artery disease, and medication use. Ethical approval was obtained for this study, and the requirement for informed consent was waived due to the retrospective observational nature of the study. (North West – Greater Manchester South Research Ethics Committee; reference number: 21/NW/0182).

### Echocardiography

All patients underwent comprehensive two-dimensional transthoracic echocardiography in the echocardiography laboratory at Barts Heart Centre using Vivid E9 or E95 scanners (GE Healthcare, Milwaukee, WI, USA) by accredited physiologists according to American Society of Echocardiography guidelines [8, 9]. Left ventricular volumes and ejection fraction were assessed using Simpson’s biplane method. LA volume was measured using the biplane disc summation method. The RV focussed apical view was used to measure RV end-systolic and diastolic area and calculate fractional area change. Right atrial area was measured on the apical four chamber view at end-systole. Mitral and tricuspid regurgitation was graded as none/trivial, mild, moderate or severe based on an integrated approach using regurgitant jet colour Doppler area, vena contracta and proximal isovelocity surface area and continuous wave Doppler profiles. Pulmonary artery systolic pressure was estimated using the tricuspid continuous wave Doppler signal and inferior vena cava size and collapsibility.

### Strain analysis

Strain measurements were performed using vendor-specific speckle-tracking software (EchoPAC version 203, GE Medical Systems, Horten, Norway). Offline strain analysis was performed retrospectively by an experienced observer blinded to clinical outcomes. Intra-observer reproducibility was assessed in 20 randomly selected studies by repeated measurements performed by the same observer blinded to the original results. Left ventricular global longitudinal strain (GLS) was obtained from apical two, three, and four-chamber views by manual tracing of the LV endocardial border. RV longitudinal strain was measured from the RV-focused apical four-chamber view.

Left atrial strain was assessed from apical two and four-chamber views, while right atrial strain was measured from the apical four-chamber view. Endocardial borders were manually traced with automated region-of-interest generation and manual adjustment when required, excluding the pulmonary veins and atrial appendages. Atrial strain analysis was performed using R-wave gating.

As most patients were in atrial fibrillation, efforts were made to minimise beat-to-beat variability during strain analysis. Whenever feasible, the index-beat method was used by selecting cardiac cycles with similar preceding R–R intervals to minimise beat-to-beat variability. When this was not feasible because of rhythm irregularity or image quality, measurements were averaged over three cardiac cycles with comparable R–R intervals. Heart rate at the time of echocardiography was recorded and included in the baseline characteristics.

### Study outcomes

The primary outcome was all-cause mortality, defined as death from any cause during follow-up. Follow-up time was calculated from the date of the baseline echocardiogram until death or the end of the study period. Mortality data were collected from NHS Spine Personal Demographics Service (PDS), which is a central registry that stores definitive demographic data, including the nationally recorded date of death, thereby ensures comprehensive capture of deaths occurring both within and outside our institution.

### Statistical analysis

Categorical variables are presented as counts and percentages and were compared using the Pearson chi-square test or Fisher’s exact test, as appropriate. Continuous variables are expressed as mean ± standard deviation (SD). Normality was assessed using visual inspection of histograms and Q-Q plots together with the Shapiro-Wilk test. Continuous variables were compared using independent-samples t-tests. Kaplan-Meier survival analysis was used to compare all-cause mortality between patients with AFTR and controls, with between-group differences assessed using the log-rank test. Cox proportional hazards regression analysis was performed to identify predictors of all-cause mortality. All strain parameters were analysed as continuous variables, with hazard ratios expressed per 1%-point change in strain, no strain-based cut-off values were applied. Variables for univariable Cox regression were selected based on their clinical relevance to mortality and were chosen to represent demographic and cardiovascular risk factors, clinical disease burden, and key echocardiographic measures of TR severity, right ventricular function, and right atrial remodelling. Multivariable Cox regression was then performed to assess the independent association of RA reservoir strain with mortality, adjusting for age and sex as demographic confounders and TR Vmax as a marker of haemodynamic disease severity. Given the potential influence of concomitant mitral regurgitation on outcomes in AFTR, Cox regression analysis was repeated in the subgroup with isolated AFTR and no mitral regurgitation. All statistical analyses were performed using SPSS Statistics version 31 (IBM Corp., Armonk, NY, USA). A two-tailed p-value < 0.05 was considered statistically significant.

## Results

**Fig. 1:**
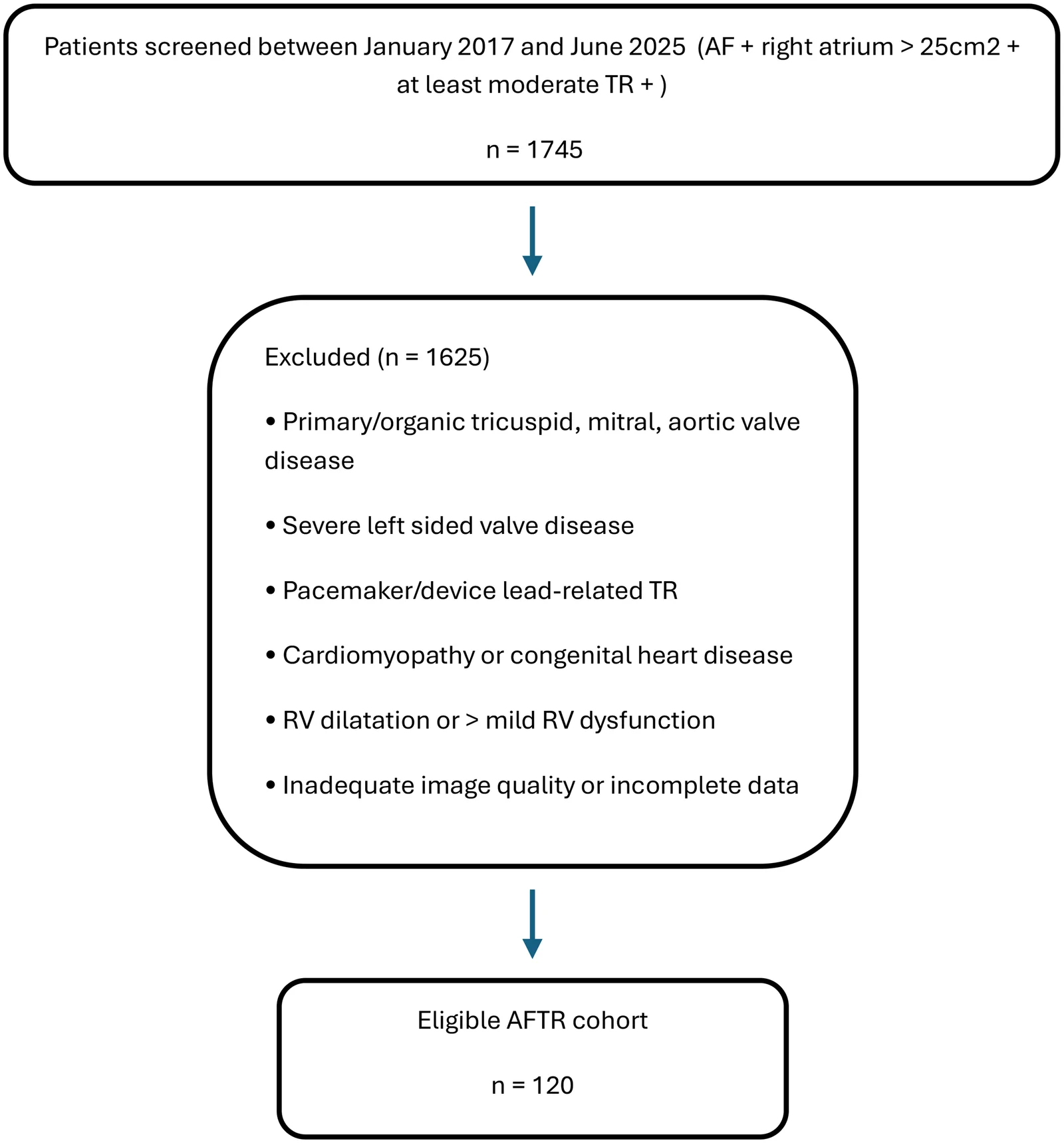
Study flow diagram

The study selection process and exclusion criteria are summarised in Fig. 1. A total of 152 patients were included in the analysis, comprising 120 patients with AFTR, and 32 controls with atrial fibrillation and mild/trivial/no mitral or tricuspid regurgitation. Among the controls, 10 (31%) had a right atrial area > 25 cm². Among patients with AFTR, 51 (43%) had isolated AFTR without any degree of MR, while 69 (57%) had concomitant atrial functional MR.

### Baseline characteristics

Baseline clinical characteristics are presented in Table 1. There were no significant differences between groups in age, body mass index, body surface area, heart rate, systolic blood pressure, or diastolic blood pressure. Patients with AFTR were more frequently female compared with controls (63.2% vs. 28.1%, *p* < 0.001). Smoking history was more common among controls (40.6% vs. 15.2%, *p* = 0.002), whereas shortness of breath was more prevalent in the AFTR group (47.7% vs. 25.8%, *p* = 0.030). There were no significant differences in the prevalence of diabetes mellitus, hypertension, hyperlipidaemia, prior myocardial infarction, family history, chest pain, palpitations, or syncope between groups. Persistent atrial fibrillation was the predominant AF subtype in both groups, accounting for 72% of the AFTR cohort and 56% of controls (*p* = 0.204). With regard to medical therapy, there were no significant differences in the use of beta-blockers, ACE inhibitors, angiotensin receptor blockers, or anticoagulation between groups. However, diuretic use was significantly more common in patients with AFTR compared with controls (55% vs. 28%, *p* = 0.007).

**Table 1:** Baseline characteristics

Variable	AFTR (*n* = 120)	Controls (*n* = 32)	*p*-value
Age, years	68 ± 26	71 ± 10	0.53
Female sex, n (%)	74 (63%)	9 (28%)	< 0.001
BMI, kg/m²	27 ± 7	27 ± 4	0.97
BSA, m²	1.7 ± 0.5	1.7 ± 0.7	0.81
Heart rate, bpm	78 ± 21	76 ± 13	0.55
Systolic BP, mmHg	135 ± 22	141 ± 20	0.21
Diastolic BP, mmHg	74 ± 13	78 ± 10	0.21
Diabetes mellitus, n (%)	29 (25%)	4 (13%)	0.13
Hypertension, n (%)	80 (68%)	19 (59%)	0.34
Hyperlipidaemia, n (%)	26 (22%)	5 (16%)	0.40
Myocardial infarction, n (%)	19 (16%)	3 (9%)	0.32
Heart failure, n (%)	32 (27%)	8 (25%)	0.83
Family history, n (%)	7 (6%)	0 (0%)	0.35
Smoking history, n (%)	17 (15%)	13 (41%)	0.002
Chest pain, n (%)	8 (7%)	2 (7%)	1.00
Palpitations, n (%)	9 (8%)	5 (16%)	0.19
Syncope, n (%)	4 (4%)	0 (0%)	0.58
Shortness of breath, n (%)	52 (48%)	8 (26%)	0.03
Atrial fibrillation subtype			0.20
New AF, n (%)	29 (24%)	11 (34%)	
Paroxysmal AF, n (%)	5 (4%)	3 (9%)	
Persistent AF, n (%)	86 (72%)	18 (56%)	
Beta-blocker, n (%)	70 (60%)	21 (66%)	0.59
ACE inhibitor, n (%)	35 (30%)	8 (25%)	0.57
Anticoagulation, n (%)	72 (62%)	25 (78%)	0.09
Diuretics, n (%)	64 (55%)	9 (28%)	0.007
Angiotensin receptor blocker, n (%)	17 (15%)	5 (16%)	0.89

Values are presented as mean ± standard deviation or n (%). Continuous variables were compared using independent-samples t-tests. Categorical variables were compared using Pearson’s chi-square test or Fisher’s exact test where appropriate. BMI, body mass index; BSA, body surface area; BP, blood pressure; AF, atrial fibrillation; ARB, angiotensin receptor blocker

### Echocardiographic characteristics

Echocardiographic characteristics are summarized in Table 2. Patients with AFTR demonstrated significantly larger atrial dimensions compared with controls, including greater LAD (4.6 ± 0.8 vs. 3.9 ± 0.8 cm, *p* < 0.001), indexed LAV (64 ± 36 vs. 44 ± 10 mL/m²,*p* < 0.001), and RA area (27 ± 10 vs. 22 ± 5 cm²,*p* < 0.001). Markers of right-sided hemodynamic burden were higher in the AFTR group, including TR Vmax (2.9 ± 0.5 vs. 2.1 ± 0.9 m/s, *p* < 0.001), RAP (7 ± 5 vs. 5 ± 4 mmHg, *p* = 0.006), and PASP (41 ± 15 vs. 26 ± 15 mmHg, *p* < 0.001).

**Table 2:** Echocardiography characteristics

Variable	AFTR overall (*n* = 120)	Controls (*n* = 32)	*p*-value
**MR severity, n (%)**			
**None/trivial**	51 (42.5)	32 (100)	-
**Mild**	36 (30.0)	0	
**Moderate**	29 (24.2)	0	
**Severe**	4 (3.3)	0	
LAD, cm	4.6 ± 0.8	3.9 ± 0.8	< 0.001
LAV indexed, ml/m²	64 ± 36	44 ± 10	< 0.001
RA area, cm²	27 ± 10	22 ± 5	< 0.001
LVIDd indexed, cm/m²	2.6 ± 0.4	2.5 ± 0.2	0.11
LVIDs indexed, cm/m²	1.7 ± 0.4	1.6 ± 0.3	0.12
LVEF by Simpson, %	61 ± 9	59 ± 8	0.37
RV FAC, %	37 ± 9	39 ± 5	0.11
TAPSE, cm	1.8 ± 0.5	1.9 ± 0.5	0.18
TR Vmax, m/s	2.9 ± 0.5	2.1 ± 0.9	< 0.001
RAP, mmHg	7 ± 5	5 ± 4	0.006
PASP, mmHg	41 ± 15	26 ± 15	< 0.001
LV septal E′, m/s	0.08 ± 0.03	0.08 ± 0.02	0.82
LV lateral E′, m/s	0.11 ± 0.04	0.11 ± 0.04	0.68
LV average E′, m/s	0.09 ± 0.03	0.30 ± 1.09	0.32
RV S′, m/s	0.11 ± 0.03	0.12 ± 0.03	0.01
E/E′ septal	14 ± 8	12 ± 4	0.02
E/E′ average	12 ± 6	9 ± 4	0.03
GLS average, %	-15 ± 3	-15 ± 8	0.72
RV longitudinal strain, %	-16 ± 4	-19 ± 5	0.01
Reservoir LA strain, %	9 ± 4	11 ± 5	0.06
Reservoir RA strain, %	10 ± 5	16 ± 8	0.001

Values are presented as mean ± standard deviation. Continuous variables were compared using independent-samples t-tests. LAD, left atrial diameter; LAV, left atrial volume; RA, right atrial; LVIDd, left ventricular internal diameter in diastole; LVIDs, left ventricular internal diameter in systole; LVEF, left ventricular ejection fraction; RV FAC, right ventricular fractional area change; TAPSE, tricuspid annular plane systolic excursion; TR, tricuspid regurgitation; RAP, right atrial pressure; PASP, pulmonary artery systolic pressure; GLS, global longitudinal strain

There were no significant differences in indexed LV dimensions, LVEF, RV FAC, or TAPSE between groups. Similarly, LV septal and lateral E′ velocities were comparable. RV S′ velocity was mildly lower in patients with AFTR (0.11 ± 0.03 vs. 0.12 ± 0.03 m/s, *p* = 0.014), while E/E′ septal and average ratios were significantly higher compared with controls.

Strain analysis demonstrated impaired RV longitudinal strain in the AFTR group compared with controls (-16 ± 4 vs. -19 ± 5, *p* = 0.010). Reservoir RA strain was also significantly reduced (10 ± 5 vs. 16 ± 8, *p* = 0.001), whereas GLS and reservoir LA strain did not differ significantly between groups. Intra-observer reproducibility for RA reservoir strain was excellent (ICC 0.97, 95% CI 0.91–0.99, *p* < 0.001).

### Predictors of all-cause mortality

**Fig. 2:**
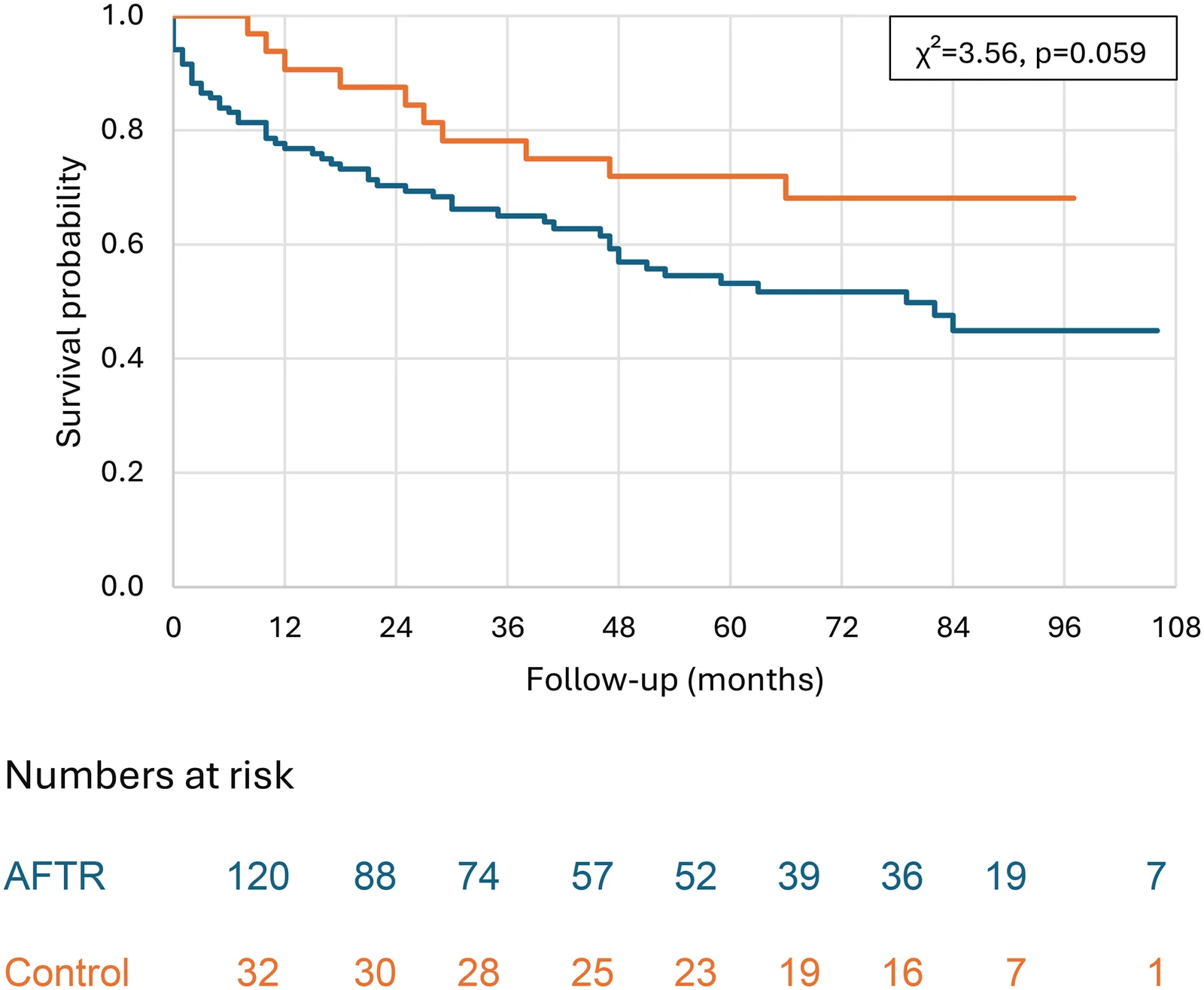
Kaplan-Meier survival curves for patients with atrial functional tricuspid regurgitation and controls. Kaplan-Meier estimates of all-cause survival in patients with atrial functional tricuspid regurgitation (AFTR; blue, *n* = 120) and controls with atrial fibrillation without significant mitral or tricuspid regurgitation (orange, *n* = 32). Numbers at risk are shown below the graph. Survival distributions were compared using the log-rank test (χ² [1] = 3.56, *P* = 0.059)

During a median follow-up of 6.3 years (95% CI 6.1–6.6 years), 63 deaths (41%) occurred in the overall study population. Of these, 53 (44%) deaths occurred among patients with AFTR and 10 (31%) among controls. Kaplan-Meier analysis demonstrated lower survival in patients with AFTR compared with controls, although the between-group difference did not reach statistical significance (log-rank *p* = 0.059, Fig. 2). In the overall cohort, AFTR was associated with a numerically higher hazard of all-cause mortality compared with controls on univariable Cox regression (HR 1.89, 95% CI 0.96–3.72, *p* = 0.065).

After exclusion of the control group, 53 deaths (44%) occurred among patients with AFTR and were included in the AFTR-specific survival analyses. Cox proportional hazards regression analysis was performed to identify predictors of all-cause mortality (Table 3). In univariable analysis, older age (HR 1.02, 95% CI 1.00–1.04, *p* = 0.016), higher TR Vmax (HR 1.59, 95% CI 1.07–2.35, *p* = 0.020), and lower reservoir RA strain (HR 0.90, 95% CI 0.84–0.96, *p* = 0.003) were significantly associated with increased mortality risk. Myocardial infarction showed a borderline association with mortality (HR 1.90, 95% CI 0.99–3.64, *p* = 0.054), whereas diabetes, smoking, heart failure history, diuretic use, RV fractional area change, RV longitudinal strain, RA area, and female sex were not significantly associated with all-cause mortality (Table 3).

**Table 3:** Cox regression in overall atrial functional tricuspid regurgitation (*n* = 120)

Variable	Univariable HR (95% CI)	*p*-value	Multivariable HR (95% CI)	*p*-value
AFTR vs. control*	1.89 (0.96–3.72)	0.065	-	-
Age	1.02 (1.00–1.04)	0.02	1.01 (1.00–1.03)	0.11
Female sex	1.06 (0.60–1.89)	0.84	0.65 (0.33–1.29)	0.22
Diabetes	1.66 (0.92–3.01)	0.092	–	–
Smoking	1.35 (0.92–1.98)	0.125	–	–
HF	0.76 (0.40–1.45)	0.410	–	–
MI	1.90 (0.99–3.64)	0.054	–	–
Diuretic use	1.04 (0.60–1.80)	0.878	–	–
TR Vmax	1.59 (1.07–2.35)	0.02	1.38 (0.85–2.24)	0.19
RV FAC	0.97 (0.94–1.01)	0.158	–	–
RV longitudinal strain	1.07 (0.99–1.16)	0.09	-	-
RA area	1.01 (0.99–1.04)	0.439	–	–
Reservoir RA strain	0.90 (0.84–0.96)	0.003	0.88 (0.81–0.95)	< 0.001

*AFTR versus control was assessed in the overall cohort (*n* = 152), all remaining Cox regression analyses were restricted to patients with AFTR (*n* = 120).Values are presented as hazard ratio (HR) with 95% confidence intervals (CI). Univariable and multivariable associations with all-cause mortality were assessed using Cox proportional hazards regression analysis. The multivariable model was adjusted for age, sex, and TR Vmax. HR, hazard ratio; CI, confidence interval; DM, diabetes mellitus; HF, heart failure; MI, myocardial infarction; TR, tricuspid regurgitation; RV, right ventricular; FAC, fractional area change; RA, right atrial

After adjustment for clinically relevant covariates including age, sex, and TR Vmax, reservoir RA strain remained the only independent predictor of all-cause mortality (adjusted HR 0.88, 95% CI 0.81–0.95, *p* < 0.001).

To assess whether concomitant mitral regurgitation influenced the observed associations, Cox regression analysis was repeated in patients with isolated AFTR without any degree of MR (Table 4). In univariable analysis, older age was associated with increased mortality risk (HR 1.05, 95% CI 1.01–1.10, *p* = 0.025), and lower reservoir RA strain was also associated with worse survival. (HR 0.91, 95% CI 0.83-1.00, *p* = 0.045). Female sex, TR Vmax, and RV longitudinal strain were not significantly associated with mortality.

**Table 4:** Cox regression in standalone atrial functional tricuspid regurgitation (*n* = 51)

Variable	Univariable HR (95% CI)	*p*-value	Multivariable HR (95% CI)	*p*-value
Age	1.05 (1.01–1.10)	0.03	1.06 (0.99–1.12)	0.08
Female sex	0.62 (0.23–1.68)	0.35	0.30 (0.09–1.05)	0.06
TR Vmax	1.63 (0.58–4.62)	0.36	0.63 (0.15–2.60)	0.52
RV longitudinal strain	1.01 (0.89–1.15)	0.85		
Reservoir RA strain	0.91 (0.83–1.00)	0.045	0.86 (0.77–0.96)	0.005

Values are presented as hazard ratio (HR) with 95% confidence intervals (CI). Univariable and multivariable associations with all-cause mortality were assessed using Cox proportional hazards regression analysis. The multivariable model was adjusted for age, sex, and TR Vmax. HR, hazard ratio; CI, confidence interval; TR, tricuspid regurgitation; RA, right atrial

A multivariable Cox regression model adjusted for age, sex, and TR Vmax demonstrated that reservoir RA strain remained independently associated with all-cause mortality in patients with isolated AFTR (adjusted HR 0.86, 95% CI 0.77–0.96, *p* = 0.005).The magnitude of this association was similar to that observed in the overall AFTR cohort (adjusted HR 0.88, 95% CI 0.81–0.95), although the confidence interval was wider in the isolated-AFTR subgroup, reflecting the smaller sample size.

## Discussion

In this study, we provide a comprehensive evaluation of cardiac structure, deformation, and clinical outcomes in patients with AFTR. Several important findings emerged. First, patients with AFTR demonstrated marked biatrial remodelling with significant impairment in right atrial reservoir function compared with controls despite preserved conventional biventricular systolic function. Second, impaired RA reservoir strain was independently associated with all-cause mortality, even after adjustment for clinically relevant variables including age, sex, and TR severity. Third, this association persisted in the subgroup with isolated AFTR without concomitant mitral regurgitation, suggesting that the prognostic significance of RA dysfunction is intrinsic to the AFTR phenotype rather than driven by coexisting AFMR. These findings highlight the central role of atrial cardiomyopathy and right atrial dysfunction in AFTR and suggest a potential role for atrial strain imaging in risk stratification.

### Right atrial remodelling and the AFTR phenotype

AFTR represents a distinct pathophysiological entity caused primarily by atrial remodelling rather than primary leaflet pathology or ventricular systolic dysfunction [5]. In our cohort, patients with AFTR demonstrated significantly larger atrial dimensions compared with controls, including increased left atrial diameter, indexed left atrial volume, and right atrial area. These findings support the concept that long-standing atrial fibrillation promotes progressive biatrial remodelling, leading to annular dilatation and functional valvular incompetence [10, 11].

Beyond structural enlargement, patients with AFTR also demonstrated significant impairment in RA reservoir strain, suggesting functional atrial dysfunction beyond chamber dilatation alone. Atrial strain impairment has previously been linked to atrial fibrosis and adverse atrial remodelling in atrial fibrillation populations, supporting its role as a marker of atrial cardiomyopathy [12, 13]. The marked reduction in RA reservoir strain observed in our cohort likely reflects reduced atrial compliance and impaired reservoir function secondary to chronic right atrial remodelling. Collectively, these findings support the concept that AFTR is primarily a manifestation of advanced atrial cardiomyopathy rather than ventricular disease.

### Prognostic value of right atrial function

A major finding of this study is the prognostic importance of RA reservoir strain in AFTR. Reduced RA reservoir strain was independently associated with all-cause mortality in both the overall AFTR cohort and the isolated AFTR subgroup without concomitant MR. The magnitude of the association was similar in the overall cohort (adjusted HR 0.88, 95%CI 0.81–0.95) and the isolated-AFTR subgroup (adjusted HR 0.86, 95%CI 0.77–0.96), indicating that excluding patients with concomitant AFMR did not weaken the association. The wider confidence interval in the isolated subgroup likely reflects its smaller sample size and reduced precision. These findings suggest that the association was not solely driven by concomitant AFMR, although a contribution from AFMR cannot be excluded without a formal interaction analysis. Importantly, this association remained significant after adjustment for age, sex, and TR severity, suggesting that RA dysfunction provides prognostic information beyond conventional clinical and echocardiographic parameters. Galloo et al. previously demonstrated that impaired RA reservoir strain was independently associated with mortality in patients with significant TR, although the majority of patients had ventricular or secondary forms of TR [14]. Our findings extend these observations specifically to AFTR and suggest that RA dysfunction itself may represent a central driver of adverse outcomes in atrial functional valve disease. Reduced RA reservoir function likely reflects advanced atrial fibrosis and impaired atrial compliance, limiting the ability of the right atrium to accommodate venous return and contributing to systemic venous congestion and progressive right-sided heart failure physiology. Although patients with AFTR demonstrated higher pulmonary pressures and mildly impaired RV deformation parameters, conventional RV systolic parameters including TAPSE and RV fractional area change remained preserved. These findings support the concept that AFTR may be associated with early right-sided haemodynamic and functional changes despite preserved conventional RV systolic function.

### Clinical implications

Current assessment of AFTR primarily relies on TR severity and conventional echocardiographic parameters. However, these measures may not fully reflect the extent of underlying atrial cardiomyopathy and right-sided functional impairment. Our findings suggest that RA deformation imaging may provide additional insight into disease severity in AFTR, particularly in patients with preserved conventional RV systolic function.

As transcatheter therapies for tricuspid valve disease continue to evolve, improved phenotypic characterization and risk stratification of AFTR patients will become increasingly important. Further prospective studies are required to determine whether RA strain may help guide clinical surveillance, timing of intervention, or selection of patients most likely to benefit from emerging therapies. Medical strategies that may impact on the course of atrial disease should be investigated in the future. A recent meta-analysis reported a lower incidence of AF with SGLT2 inhibitors across several cardiovascular populations [15], raising the question of whether such treatment could also influence atrial remodelling and the natural history of AFTR. This possibility has not yet been established and requires prospective investigation.

### Limitations

This was a retrospective single-centre study and may therefore be subject to selection bias. Strain analysis was performed using a single vendor-specific platform, which may limit generalizability across different software vendors. The beat-selection method was not recorded for each study, preventing comparison between index-beat selection and three-cycle averaging and potentially introducing variability into the strain measurements. Although diuretic use was assessed, data on diuretic dose, renal function, and natriuretic peptide levels were unavailable. Therefore, residual confounding by baseline heart-failure severity cannot be excluded. Only all-cause mortality data were available, as cause-specific mortality and heart-failure hospitalisation data were not recorded. This limited our ability to determine whether the association between RA reservoir strain and mortality was driven specifically by cardiovascular outcomes. Finally, the sample size for the survival analysis was modest, and the findings should therefore be considered hypothesis-generating until confirmed in larger prospective cohorts.

## Conclusion

AFTR represents a distinct atrial cardiomyopathy phenotype characterized by marked biatrial remodelling and impaired right atrial function. Reduced RA reservoir strain was independently associated with mortality, even in isolated AFTR without concomitant mitral regurgitation. RA deformation imaging may therefore provide valuable prognostic value and improve risk stratification in patients with AFTR.

## Data Availability

The datasets generated and/or analysed during the current study are not publicly available due to patient confidentiality regulations but are available from the corresponding author on reasonable request.

## Abbreviations

AF: Atrial fibrillation
AFTR: Atrial functional tricuspid regurgitation
CI: Confidence interval
FAC: Fractional area change
HR: Hazard ratio
LA: Left atrial/left atrium
LV: Left ventricular/left ventricle
LVEF: Left ventricular ejection fraction
PASP: Pulmonary artery systolic pressure
RA: Right atrial/right atrium
RV: Right ventricular/right ventricle
TAPSE: Tricuspid annular plane systolic excursion
TR: Tricuspid regurgitation
